# Report of Operation for Multiple Abscess of Pelvis with Slough of Six Inches of Colon; Subsequent Anastamosis with Connell Suture; Recovery

**Published:** 1907-07

**Authors:** E. C. Davis

**Affiliations:** Atlanta, Ga., Professor of Gynecology and Obstetrics, Atlanta School of Medicine; Member American Medical Association, Medical Association of Georgia, Fulton County Medical Society, Gynecologist to Tapernacle Infirmary and Presbyterian Hospital, Surgeon to McViccar Hospital for Colored Women


### REPORT OF OPERATION FOR MULTIPLE ABSCESS OF PELVIS WITH SLOUGH OF SIX INCHES OF COLON; SUBSEQUENT ANASTAMOSIS WITH CONNELL SUTURE; RECOVERY.*

By C. E. Davis, M. D., Atlanta, Ga., Professor of Gynecology and Obstetrics, Atlanta School of Medicine; Member American Medical Association, Medical Association of Georgia, Fulton County Medical Society, Gynecologist to Tapernacle Infirmary and
ty Medical Society, Gymeologist to Tabernacle Infirmary and Presbyterian Hospital, Surgeon to McViccar Hospital for Colored Women.

The proper handling of suppurating conditions is one of the most difficult of all surgical problems, and one which taxes the ingenuity and skill to the highest possible degree; when we add to this the liberation of numerous adhesions and devitalization of tissues to such an extent that sloughing ensues, we add materially to the difficulties of the problem and to the danger of the operative procedures. The case which I wish to report accentuates these difficulties markedly, and possesses a peculiar interest from several standpoints.

Mrs. C.—Multipara, about two years previous had a violent attack of pelvis peritonitis which had recurred at frequent intervals, until her health was completely undermined and she had become practically an invalid. On examining her I found a retrodisplaced fixed uterus, lacerated cervix and a mass on both sides and behind the uterus. This mass extended high up and was very sensitive to the touch. The uterus was soft and boggy to the touch with signs of chronic metritis. She was sent to the Tabernacle Infirmary and a specimen of urine carefully examined showing marked glycosuria. She was then put on a careful regulated diet and at the expiration of twelve days the urine had become normal, and she insisted upon

*Read before the Fulton County Medical Society, June 6, 1907.

having the operation performed, stating that she would assume all risk as life in her present condition was not worth living. After the usual preparations the operation was begun Sep. 8, 1906. A free incision was made in median line and the intestines carefull protected with gauze pads, a mass of adhesions was found and two extensive abscesses, the one on the right side taking the course of the ureter on that side and so firmly attached to parietal peritoneum that when dissected up the ureter was bared for a considerable distance. This was promptly covered over, by suturing the torn peritoneum with cat gut. On the left side a more difficult problem presented itself, for here the colon just above the sigmoid had become firmly agglutinated to the abscess on this side and on endeavoring to separate this the gut was denuded of its peritoneum for a distance of about six inches. The uterus was friable and soft, appearing thoroughly septic and was removed. The patient now became seriously shocked and the anesthetist began to urge haste, so while judgment dictated under ordinarily favorable conditions a resection of the gut at this time, was afraid she would succumb on the table, so sutured over the raw surfaces in peritoneum, trusting that enough vascularity still remained to insure proper nourishment of the tissues, drainage was placed through vagina and also through abdominal incision using gauze. Patient was put to bed in very precarious condition and for five days pulse ranged from an inability to count it to 135 per minute. At the expiration of the fifth day gauze was removed, bowels had acted several times, though condition of patient had still but slightly improved- Stools became involuntary, patient slightly desirous. On the tenth day a fecal fistula developed through vagina and the denuded portion of gut was cast off through the vagina intact. After this occurred the patient began to improve, pulse coming down and getting some stronger. A very singular feature about this case was that nature endeavored to make an anastamosis, as after some days the stools passed through their normal route and we hoped for a restoration without another operation. This was of but short duration, for stools soon ceased to pass through anus and the vagina was constantly soiled by flow of fecal discharge. Patient was too weak to undergo any additional operation now, and was sent home to recuperate. She improved in general health rapidly and returned November 28th for the completion of the operation. The operation was performed two days after admission, great care being given to preliminary preparation. Incision was again made in median line as in previous operation. Was much gratified to note the absence of adhesions at former operative site. We now endeavored to find the two ends of separated gut, found the one attached to vagina without difficulty and then passing finger behind and to left side found the other end of gut. These were brought together and sutured with Connell mattress suture, fortified with Cushings' continuous suture, using chromic cat gut for first and plain cat gut for second, the vaginal opening was closed except for small space, through which was passed a strip of gauze and left in contact with sutured gut. The abdominal incision was closed. The operation was very tedious and again patient was put to bed in a most critical condition.

For five days pulse was often too weak to count, on fifth day a sterile olive oil enema was administered and several hours afterwards an ounce of Castor oil given by mouth. This yielded no result so she was given calomel regularly. She then became nauseated and vomited fecal matter, the rectum was irrigated, using Wales bougie and she soon passed fecal matter, some coming through vagina and some through rectum. The rectum was now regularly dilated and kept clean, vagina was irrigated and after a few days the fistulous margin touched with strong solution of nitrate of silver. She began to improve almost as soon as bowels acted and under this method of treatment vaginal fistula closed entirely and she is today apparently well with bowels acting normally, having gained about thirty pounds. Urine has become normal.

This case illustrates several very interesting points, viz.—Is there any co-relation between chronic pelvic suppuration and glycosuria?

Second. Nature may cast off large piece of intestine and recovery ensue.

Third. Nature endeavors to make an anastamosis, pointing out what we ought to do.

Fourth. The utilization of suture material in place of mechanical devices to approximate the ends of the intestines.

Since this report was contemplated I have been privileged to resect large and small intestine using same suture with perfectly satisfactory results and no leakage.

DISCUSSION:—Dr. Cyrus Strickler said that his own experience was limited to one case and in this the condition was very critical, consequently he had used a Murphy button but believed the suture was better.

Dr. J. N. Ellis had found the Murphy button very useful where time was an important factor.

Dr. E. G. Jones asked if Dr. Davis had used the continuous suture. He had found the Myomiban method very valuable.

Dr. Davis (closing) endorsed what Dr. Ellis had said regarding the Murphy button, as he had used it on several occasions, in one of which it was passed on the 96th day. He did not think it could be easily applied where the parts to be united were deep in the pelvis. Answering Dr. Jones question he said that he used the interrupted suture and the Moynnihan and Cushing continuous suture.

